# Reliability of attention bias and attention bias variability to climate change images in the dot-probe task

**DOI:** 10.3389/fpsyg.2022.1021858

**Published:** 2023-01-12

**Authors:** Joshua M. Carlson, Lin Fang, Caleb Coughtry-Carpenter, John Foley

**Affiliations:** Department of Psychological Science, Northern Michigan University, Marquette, MI, United States

**Keywords:** dot-probe, reliability, climate change, attention bias, attention bias variability

## Abstract

Climate change is one of the most pressing issues of the 21st century, which is perhaps why information about climate change has been found to capture observers’ attention. One of the most common ways of assessing individual differences in attentional processing of climate change information is through the use of reaction time difference scores. However, reaction time-based difference scores have come under scrutiny for their low reliability. Given that a primary goal of the field is to link individual differences in attention processing to participant variables (e.g., environmental attitudes), we assessed the reliability of reaction time-based measures of attention processing of climate change information utilizing an existing dataset with three variations of the dot-probe task. Across all three samples, difference score-based measures of attentional bias were generally uncorrelated across task blocks (*r* = −0.25 to 0.31). We also assessed the reliability of newer attention bias variability measures that are thought to capture dynamic shifts in attention toward and away from salient information. Although these measures were initially found to be correlated across task blocks (*r* = 0.17–0.67), they also tended to be highly correlated with general reaction time variability (*r* = 0.49–0.83). When controlling for general reaction time variability, the correlations across task blocks for attention bias variability were much weaker and generally nonsignificant (*r* = −0.25 to 0.33). Furthermore, these measures were unrelated to pro-environmental disposition indicating poor predictive validity. In short, reaction time-based measures of attentional processing (including difference score and variability-based approaches) have unacceptably low levels of reliability and are therefore unsuitable for capturing individual differences in attentional bias to climate change information.

## 1. Introduction

Anthropogenic climate change is one of the most serious problems facing the global community (Pecl et al., 2017; Tollefson, 2019, 2020). Information about climate change should therefore demand individuals’ attention (Luo and Zhao, 2021). Yet, only recently has research explored the extent to which climate change related information captures attention. Initial research using climate change relevant images of environmental damage found that these images captured the attention of individuals with pro-environmental attitudes (Beattie and McGuire, 2012). Follow up studies have generally supported the finding that climate change-relevant (or other environmentally harmful) objects capture attention in individuals with pro-environmental dispositions (Sollberger et al., 2017; Carlson et al., 2019b; Meis-Harris et al., 2021). In addition, words related to climate change (Whitman et al., 2018) and graphical information of climate change (Luo and Zhao, 2019) capture attention in politically liberal individuals more concerned with climate change. Thus, there is emerging evidence that climate change relevant information captures observers’ attention—what can be referred to as an *attentional bias* for climate change or environmentally relevant information. Such findings may offer insight into how best disseminate information about climate change that is attention grabbing in such a way as to promote large scale societal changes.

Research assessing the attentional capture of environmentally relevant stimuli has primarily used reaction time (RT; Carlson et al., 2019b, 2020; Meis-Harris et al., 2021) and eye tracking (Beattie and McGuire, 2012; Sollberger et al., 2017; Luo and Zhao, 2019) based measures. RT measures of attentional bias are typically calculated using a difference score (e.g., the difference in RTs between conditions where attention is facilitated vs. not facilitated). Broadly speaking, RT-based difference scores have come under scrutiny for low internal and/or test–retest reliability (Hedge et al., 2018; Goodhew and Edwards, 2019). For individual differences (i.e., correlational) research, between subject variability is necessary and needs to consistently/reliably measure the construct of interest. Given that one of the broad goals in the newly developing field of environmental attention bias research is to link variability in attentional processing to individual differences such as pro-environmental disposition (Beattie and McGuire, 2012; Sollberger et al., 2017; Carlson et al., 2019b; Meis-Harris et al., 2021) and political orientation (Whitman et al., 2018; Luo and Zhao, 2019), it is important to assess the reliability of environmental attention bias measures. However, reliability estimates for attention bias measures are rarely reported in the literature.

Given the low reliability of RT difference score-based estimates of attentional bias, the field of experimental psychopathology (where attentional bias is often linked to affective disorders/traits) sought to improve upon traditional (difference score-based) attention bias measures. As a result, innovative attention bias variability (ABV) measures were developed, which are thought to capture dynamic shifts of attention with alternating periods of attentional focus toward and away from affective information (Iacoviello et al., 2014; Zvielli et al., 2015). Early research using ABV measures found that they were more reliable than the traditional approach (Naim et al., 2015; Price et al., 2015; Davis et al., 2016; Rodebaugh et al., 2016; Zvielli et al., 2016; Molloy and Anderson, 2020). However, subsequent work has shown that general RT variability and mean RT speed influence measures of ABV and when controlled for significantly reduce their reliability (Kruijt et al., 2016; Carlson and Fang, 2020; Carlson et al., 2022a). ABV measures have not been used in the field of environmental attention bias. However, if found to be reliable in this context, they could be useful measures of attentional bias to environmental information.

Given that RT measures are commonly used in environmental attention bias research, and a goal of this research is often to link variability in attentional biases to relevant individual differences, we sought to assess the reliability of both traditional attention bias and innovative ABV measures. To meet this end, we utilized three existing datasets from previously published research utilizing the dot-probe task to assess attentional bias to emotionally positive and negative climate change relevant images (Carlson et al., 2020). We computed the correlation of attention bias measures for emotionally positive and negative climate change relevant images across blocks in the dot-probe tasks to assess the reliability of these measures. Based on previous research using non-environmental stimuli (e.g., threat or food related images; Carlson and Fang, 2020; Vervoort et al., 2021), we hypothesized that RT measures of attentional bias to climate change information would not be reliable and therefore unsuitable for individual differences research.

## 2. Materials and methods

### 2.1. Participants

This report included 177 participants from three separate samples. Sample one contained 58 (female = 47) individuals between the ages of 18–34 (*M* = 21.00, *SD* = 3.71). Sample two included 59 (female = 45) individuals 18–38 years old (*M* = 20.41, *SD* = 3.69). Sample three was comprised of 60 individuals (female = 52) 18–36 years old (*M* = 20.68, *SD* = 3.83). With *N* ≥ 58, this study was powered to detect correlations of *r* ≥ 0.35 (with each sample at ⍺ = 0.05, and power = 0.80) and therefore able to detect reliabilities considered to be unacceptably low. Across all three samples, participants provided informed written consent and received course credit (in undergraduate psychology courses) for their participation. The study was approved by the Northern Michigan University (NMU) Institutional Review Board (IRB; HS16-768).

### 2.2. Dot-probe task

**Figure 1:**
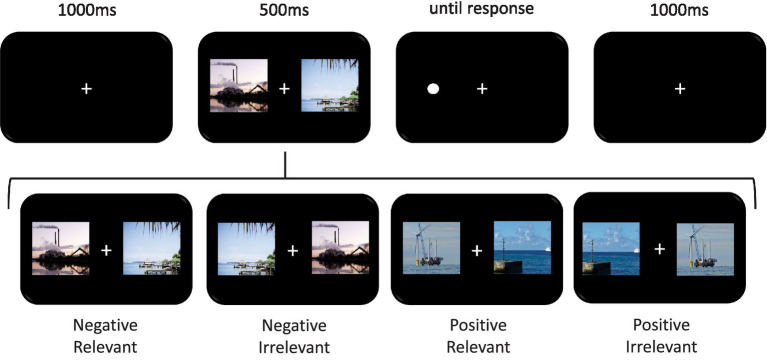
Trial structure of the dot-probe task of attentional bias. First, each trial started with a 1,000 ms white fixation cue (+) in the center of a black background. Second, two images were simultaneously presented to the left and right side of the fixation cue for 500 ms. All trials contained a climate relevant and a climate irrelevant image pair. These images each extended 10° × 12° of the visual angle and were separated by 14.5° of the visual angle. Third, a target dot appeared immediately after the images were removed and remained on the screen until a response was recorded. Fourth and finally, a 1,000 ms intertrial interval separated trials. Participants were seated 59 cm from the screen and instructed to focus on the central fixation cue throughout each trial while using their peripheral vision to locate the target dot as quickly as possible. An E-Prime serial response box was used to indicate left and right sided targets by (respectively) pressing the “1” button with their right index finger and the “2” button with their right middle finger. Faster reaction times to targets occurring at locations preceded by a climate change relevant image indicates attentional bias for the climate change relevant image over the irrelevant image.

Each sample utilized a modified dot-probe task (MacLeod et al., 1986; MacLeod and Mathews, 1988) with climate change relevant images. The details of the specific images and dot-probe tasks used in this report have been previously published (Carlson et al., 2020). Briefly, images used in each experiment were selected from the affective images of climate change database (https://affectiveclimateimages.weebly.com; Lehman et al., 2019).^1^ The database contains a total of 320 digital images rated on their emotional valence (1 unpleasant to 9 pleasant), emotional arousal (1 calm to 9 exciting), and relevance (1 least relevant to 9 most relevant) to climate change. All tasks were programmed in E-Prime (Psychology Software Tools, Pittsburg, PA) and displayed on a 60 Hz 16′′ LCD computer monitor. All variants of the task used the same general sequence of events, which are depicted and summarized in Figure 1. It should be noted that for relatively simplistic and universal emotional stimuli (such as facial expressions), shorter (<300 ms) interstimulus intervals result in more robust bias effects (Torrence et al., 2017), greater reliability (Chapman et al., 2019), and a stronger association with anxiety (Bantin et al., 2016). However, informal pilot testing in our lab lead to the conclusion that the complex scenes of climate change related information used here would need longer display times for the content to be processed and therefore a stimulus duration of 500 ms was used here. As previously reported, this stimulus duration has been found to elicit attention bias effects for climate change images in the dot-probe task (Carlson et al., 2019b, 2020).

Climate-relevant and climate-irrelevant images were randomly presented to the left visual field or right visual field for each participant. There were an equal number of trials with the target dot occurring on the same side of the screen as the climate change-relevant image and on the same side as the climate change-irrelevant image (see below for more details of each specific sample). Faster reaction times (RTs) to targets occurring at the climate-relevant location (i.e., traditionally referred to as congruent trials in the dot-probe literature) compared to climate-irrelevant location (i.e., incongruent trials) are considered representative of attentional bias (MacLeod et al., 1986; MacLeod and Mathews, 1988). At the conclusion of each block, participants received feedback about their overall accuracy and reaction time to encourage accurate rapid responses. The specific design of each sample is summarized below.

Sample one utilized a 2 × 2 (emotional valence × relevance of the target location) factorial design and consisted of 3 blocks of 120 trials with 30 in each cell: positive relevant, positive irrelevant, negative relevant, & negative irrelevant. This yielded 360 total trials, with 90 trials in each cell. Positive images included windmills and solar panels, whereas negative images included industrial air pollution, melting ice, and natural disasters.

Sample two utilized a 2 × 3 factorial design with location relevancy (relevant vs. irrelevant) × image type (cause vs. effect vs. solution) as the independent variables. The dot-probe task used in sample 2 contained 3 blocks of 180 trials with 30 in each cell: yielding 540 total trials, with 90 trials in each cell type. Causes included images of industrial air pollution and deforestation. Effects included images of melting ice and natural disasters. Solutions included images of windmills and solar panels.

Sample three utilized a 2 × 2 factorial design with location relevancy (relevant vs. irrelevant) and image type (cause vs. effect) as the independent variables. The dot-probe task in sample 3 consisted of 3 blocks of 144 trials with 36 trials in each cell: yielding 432 total trials, with 108 trials in each cell type. Cause and effect images included the same types of stimuli used in sample 2.

### 2.3. Data reduction and analysis procedures

**Figure 2:**
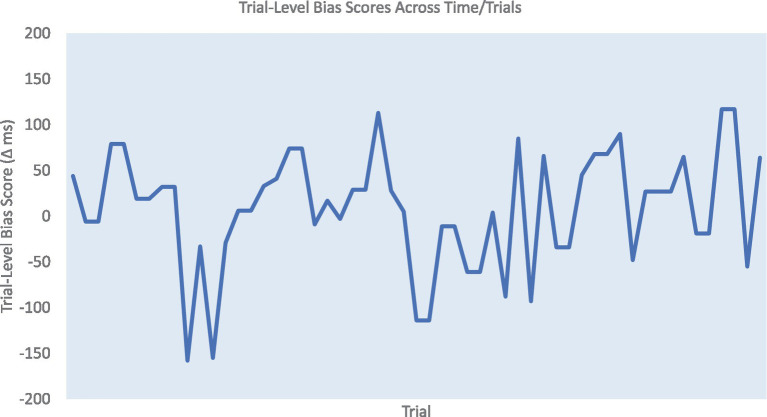
An example of trial level bias scores (TLBSs) of one block in the dot-probe task. Attention bias variability (ABV) is computed as the summed distance between succeeding TLBSs divided by the total number TLBSs.

Consistent with previous research (Torrence et al., 2017; Carlson et al., 2019a), we only included correct responses between 150 and 750 ms after the presence of the target in the dot-probe task to eliminate premature responses and lapses in attention (98.24% of the data was included for Sample 1, 95.38% for Sample 2, and 95.12% for Sample 3). Traditional attentional bias was defined as the difference between the mean RT of incongruent and congruent conditions (i.e., mean incongruent – congruent RT). The calculation of ABV was based on the trial-level bias score method (Zvielli et al., 2015), which has been shown to be more reliable than other ABV approaches (Molloy and Anderson, 2020). To compute the trial level bias score, each congruent trial was first paired with the closest incongruent trial with a maximum distance of 5 trials backward or forward. Similarly, each incongruent trial was paired with its closest congruent trial. Next, the trial level bias scores were obtained by subtracting the RT of congruent from incongruent trials for each pair (see Figure 2). To calculate ABV, the summed distance between succeeding trial level bias scores was divided by the total number of trial level bias scores. General RT variability (RTV) was obtained by calculating the standard deviation of RTs across all (congruent and incongruent) trials. All measures were computed separately for each block. To examine the reliability of traditional attentional bias and ABV, bivariate Pearson correlations across blocks were performed in SPSS 28. In addition, in order to control for the influence of general RTV, partial correlations across blocks were also conducted for each measurement.

## 3. Results

### 3.1. Traditional attentional bias score reliability

In each of the three independent samples analyzed here, traditional measures of attentional bias were generally uncorrelated across blocks 1–3 for different types of climate change images.^2^ These measures were also generally uncorrelated with general RTV and partial correlations controlling for RTV did not drastically alter the association between traditional measures of attentional bias across blocks. For all correlations with traditional measures of attentional bias, see Table 1 (samples 1–3, respectively).

**Table 1:** Correlations across blocks in samples 1–3.

**Sample 1**												
**Block**	**AB positive climate change images**						**AB negative climate change images**					
	**1**	**2**		**3**	**RTV**		**1**	**2**		**3**	**RTV**	
1	–	0.21		0.09	0.12		–	−0.06		−0.05	0.02	
2	*0.19*	–		0.09	0.24		*−0.06*	–		0.18	−0.14	
3	*0.07*	*0.05*		–	0.20		*−0.05*	*0.16*		–	−0.11	
	**ABV positive climate change images**						**ABV negative climate change images**					
1	–	0.31*		0.38*	0.65*		–	0.41*		0.29*	0.61*	
2	*−0.01*	–		0.45*	0.49*		*−0.03*	–		0.39*	0.70*	
3	*−0.05*	*0.20*		–	0.64*		*−0.16*	*−0.11*		–	0.64*	
**Sample 2**												
**Block**	**AB effects**				**AB causes**				**AB solutions**			
	**1**	**2**	**3**	**RTV**	**1**	**2**	**3**	**RTV**	**1**	**2**	**3**	**RTV**
1	–	−0.12	0.18	0.10	–	−0.02	0.06	−0.14	–	0.11	−0.09	0.22
2	*−0.11*	–	0.16	−0.02	*−0.01*	–	0.08	0.08	*0.11*	–	−0.25	0.01
3	*0.15*	*0.18*	–	0.43*	*0.07*	*0.08*	–	0.09	*−0.08*	*−0.25*	–	−0.04
	**ABV effects**				**ABV causes**				**ABV solutions**			
1	–	0.42*	0.17	0.66*	–	0.38*	0.23	0.52*	–	0.53*	0.48*	0.76*
2	*−0.13*	–	0.54*	0.74*	*0.10*	–	0.59*	0.60*	*0.13*	–	0.42*	0.58*
3	−0.29*	*0.24*	–	0.55*	*−0.25*	0.29*	–	0.72*	*−0.13*	*0.03*	–	0.68*
**Sample 3**												
**Block**	**AB effects**					**AB causes**						
	**1**	**2**		**3**	**RTV**	**1**		**2**	**3**	**RTV**		
1	–	0.27*		0.31*	−0.10	–		0.10	0.29*	0.15		
2	0.27*	–		0.06	0.04	*0.05*		–	0.22	0.30*		
3	0.31*	*0.06*		–	−0.01	0.26*		*0.16*	–	0.26*		
	**ABV effects**					**ABV causes**						
1	–	0.67*		0.54*	0.83*	–		0.59*	0.59*	0.81*		
2	*0.09*	–		0.66*	0.77*	*−0.16*		–	0.58*	0.81*		
3	*0.00*	0.33*		–	0.65*	*0.03*		*0.01*	–	0.71*		

*p < 0.05, Shaded/gray region with italicization on the bottom reflects partial correlations controlling for reaction time variability (RTV). AB, attention bias; ABV, attention bias variability.

### 3.2. Attention bias variability reliability

In general, across samples 1–3, ABV-based measures of attentional bias were moderately to highly correlated across blocks and moderately to highly correlated with general RTV. In partial correlations controlling for RTV, ABV-based measures only weakly correlated across blocks and in the majority of cases these correlations were no longer significant. See Table 1 for all ABV correlations for samples 1–3, respectively.

### 3.3. Attention bias variability correlations with pro-environmental disposition

The New Ecological Paradigm questionnaire (Dunlap et al., 2000) was administered to samples 2 and 3 as a measure of pro-environmental disposition. Although ABV in these samples appears to be driven by RT variability, we nevertheless assessed the degree to which these scores offer predictive validity for pro-environmental disposition.^3^ Across samples 2 and 3, ABV scores were unrelated to pro-environmental disposition (Sample 2: Cause *r* = 0.03, Effect *r* = −0.08, Solution *r* = −0.16 & Sample 3: Cause *r* = −0.02 & Effect *r* = 0.02, *p*s ≥ *0*.22). Note that in a separate sample, climate change anxiety was also unrelated to these ABV scores.^4^

## 4. Discussion

This study aimed to assess the reliability of RT-based measures of attention bias and ABV to climate change images in the dot-probe task. The results obtained here across three dot-probe tasks of attentional bias to climate change relevant images indicate that neither the traditional (RT difference score) approach nor the innovative ABV approach were reliable measures of attentional bias. The traditional approach was not consistently influenced by general RT variability, but was nevertheless unreliable. This finding is consistent with a growing body of literature using the dot-probe task in other fields (Schmukle, 2005; Staugaard, 2009; Price et al., 2015; Aday and Carlson, 2019; Chapman et al., 2019; Van Bockstaele et al., 2020). On the other hand, ABV scores initially correlated across blocks demonstrating some degree of reliability, which is consistent with prior ABV research (Naim et al., 2015; Price et al., 2015; Davis et al., 2016; Rodebaugh et al., 2016; Zvielli et al., 2016; Molloy and Anderson, 2020). Yet, when controlling for general RT variability, ABV measures were no longer correlated across blocks indicating that they likely measure general RT variability rather than attention bias behavior. Again, this finding echoes what has been reported in prior studies assessing attentional bias to threat and food related stimuli (Kruijt et al., 2016; Carlson and Fang, 2020; Vervoort et al., 2021; Carlson et al., 2022a). Finally, ABV measures were found to be unrelated to individual differences in pro-environmental orientation (and climate change anxiety)—suggesting poor predictive validity. Thus, many of the shortcomings of the RT difference score and ABV approaches reported in other fields appear to generalize to the use of environmental stimuli in the dot-probe task.

Based on these findings, we recommend that RT-based measures of attentional bias to environmental information should not be used for individual differences (or correlational) research. As much of the field of environmental attentional bias research is interested in linking variability in attentional bias to individual differences related to environmentalism or climate change concern (Beattie and McGuire, 2012; Sollberger et al., 2017; Whitman et al., 2018; Carlson et al., 2019b; Luo and Zhao, 2019; Meis-Harris et al., 2021), new/different approaches to capturing attentional bias are needed for these research objectives. As previously mentioned, eye tracking is another common approach to measuring attentional bias to climate change relevant information (Beattie and McGuire, 2012; Sollberger et al., 2017; Luo and Zhao, 2019). Some research suggests that eye-tracking measures of attention are (more) reliable (Sears et al., 2019; van Ens et al., 2019; Soleymani et al., 2020), whereas other research suggests that eye tracking may not be more reliable (Skinner et al., 2018). Therefore, future research should aim to assess the reliability of eye tracking-based measures from the paradigms used to measure environmental attentional bias. In addition, electroencephalographic measures of brain activity have been found to more reliably measure covert attention (Kappenman et al., 2014; Reutter et al., 2017) and may be appropriate for measuring environmental attentional biases.

Although the results obtained here indicate that RT-based measures in the dot-probe task are not suitable for capturing individual differences in attentional bias to climate change information, this does not preclude the use of RT-based tasks to assess the effects of experimental manipulations on attentional bias. Reliability is not required for comparisons across experimental groups/conditions, but is for individual differences research. The field needs to identify ways to increase the reliability of attention bias estimates. Reaction times start from a promising point (highly correlated across blocks: Sample 1: *r* = 0.77–0.92, Sample 2: *r* = 0.85–0.92, and Sample 3: *r* = 0.82–0.91), but data quality quickly diminishes when calculating differences scores (Hedge et al., 2018).

Initial research, based on RTs in the dot-probe task, indicates that emotionally positive images of climate change solutions capture attention to a greater extent than emotionally negative images of climate change causes and effects (Carlson et al., 2020). Future experimental research is needed to determine whether the same pattern is observed using other images as well as verbal, auditory, and multimodal information about climate change. Indeed, determining what types of climate change relevant information is best suited to capture individuals’ attention would be useful in the effective design of environmental communication related to climate change. Furthermore, identifying interventions, contextual factors, and other manipulations that can modify attention to climate change information has important implications for increasing attentional focus on climate change messaging. For example, research has shown that attention training can increase attention to climate change information (Carlson et al., 2022b). In summary, although much research on environmental attentional biases focuses on individual differences in attentional bias and our data indicate that RT-based (difference score & ABV) measures of attentional bias are unsuitable for correlational research, more experimental research is needed to better understand the variables that lead to an effective focus of attention on climate change information.

This study is not without limitation. First, the samples utilized here were primarily comprised of college-age females, and although it is unlikely that the reliability of attention bias and ABV measures differ across populations, the homogeneity of our sample(s) limits the generalization of the results. In addition, another limitation of this study is the sole use of climate change relevant images rather than other stimulus types. Although the reliability of attentional bias and ABV to other types of information (e.g., threat-related information) does not appear to be related to stimulus type (Staugaard, 2009; Carlson and Fang, 2020), it is possible that attention to climate change related information differs based on the stimulus type (e.g., images vs. words). Future research is needed to assess this possibility. Finally, although the dot-probe task is among the most common RT-based methods of assessing attentional bias, the extent to which RT-based reliability estimates of attentional bias observed here in the dot-probe task generalize to other RT-based tasks/measures is unclear. Yet, given that reliability is generally an issue for RT-based (difference score) measures (Hedge et al., 2018; Goodhew and Edwards, 2019), we do not expect these findings to be specific to the dot-probe task, but RT-based measures more broadly.

## 5. Conclusion

In summary, the present study aimed to assess the reliability of the dot-probe task using climate change relevant images. Both traditional (reaction time difference score) and innovative ABV measures were used, and both were found to lack reliability in measuring individual differences in attentional bias to climate images. These findings strongly suggest that the dot-probe task, and likely other RT difference score-based measures, are unsuitable for individual differences research assessing the correlation between participant factors, such as climate concern, and attention bias. Due to the growing body of work focusing on these and other individual differences, we argue that other measures of attention bias should be adopted for these purposes. No matter which measure of attentional bias is used, the reliability estimates of the measure should be included. Finally, RT-based cognitive tasks, such as the dot-probe, may still be appropriate for measuring differences in attention bias following various experimental interventions.

## Data availability statement

The datasets presented in this study can be found in online repositories. The names of the repository/repositories and accession number(s) can be found at: https://osf.io/E9S8P/.

## Ethics statement

The studies involving human participants were reviewed and approved by Northern Michigan University IRB. The patients/participants provided their written informed consent to participate in this study.

## Author contributions

JC designed the study. JC and LF processed and analyzed the data. JC, LF, CC-C, and JF drafted the manuscript. All authors contributed to the article and approved the submitted version.

## Conflict of interest

The authors declare that the research was conducted in the absence of any commercial or financial relationships that could be construed as a potential conflict of interest.

## Publisher’s note

All claims expressed in this article are solely those of the authors and do not necessarily represent those of their affiliated organizations, or those of the publisher, the editors and the reviewers. Any product that may be evaluated in this article, or claim that may be made by its manufacturer, is not guaranteed or endorsed by the publisher.

## Footnotes

^1^The following images were used in Sample 1: 12, 13, 29, 39, 46, 49 (positive relevant), 8, 18, 23, 20, 37, 62 (negative relevant), 178, 194, 200, 210, 221, 240, 256, 277, 280, 303, 308, and 315 (irrelevant). The following images were used in Sample 2: 12, 13, 29, 39, 46, 49 (solution relevant), 8, 18, 21, 23, 35, 42 (cause relevant), 7, 16, 19, 20, 37, 62 (effect relevant), 178, 194, 200, 210, 221, 222, 240, 243, 256, 277, 280, 281, 289, 295, 303, 308, 312, and 315 (irrelevant). Sample 3 used the same cause and effect images used in sample 2.

^2^Note that it could be argued that correlations might be weaker across blocks due to meaningful changes in bias across time. As an alterative approach, we performed odd-even split-half Pearson correlations for each condition. Split-half reliability for attentional bias to climate change solutions in Sample 2 revealed a significant negative correlation (*r* = −0.30, *p* = 0.023). All of the remaining split-half correlations were non-significant (*r* = −04 to 0.26, *ps* > 0.05).

^3^Note that the associations with pro-environmental disposition and traditional measures of attentional bias from this sample have been reported elsewhere (Carlson et al., 2020).

^4^In a separate sample (*N* = 120), we found no association between ABV and climate change anxiety for positive (*r* = 0.08, *p* = 0.39) and negative (*r* = −0.001, *p* = 0.99) images of climate change.
